# Using social media data in diabetes care: bridging the conceptual gap between health providers and the network population

**DOI:** 10.1186/s12875-022-01846-0

**Published:** 2022-09-17

**Authors:** Ru-Hsueh Wang, Yu-Wen Hong, Chia-Chun Li, Siao-Ling Li, Jenn-Long Liu, Chih-Hsing Wu, Ching-Ju Chiu

**Affiliations:** 1 Department of Family Medicine, College of Medicine, National Cheng Kung University Hospital, National Cheng Kung University, Tainan, Taiwan; 2 Institute of Gerontology, College of Medicine, National Cheng Kung University, Tainan, Taiwan; 3 Institute of Allied Health Sciences, College of Medicine, National Cheng Kung University, Tainan, Taiwan; 4 Department of Family Medicine, College of Medicine, National Cheng Kung University, Tainan, Taiwan; 5 Department of Information Management, I-Shou University, Kaohsiung, Taiwan

**Keywords:** Diabetes, OpView, Text mining, Social media, Sentiment analysis

## Abstract

**Background:**

Patients with diabetes who have poor health literacy about the disease may exhibit poor compliance and thus subsequently experience more complications. However, the conceptual gap of diabetes between health providers and the general population is still not well understood. Decoding concerns about diabetes on social media may help to close this gap.

**Methods:**

Social media data were collected from the OpView social media platform. After checking the quality of the data, we analyzed the trends in people’s discussions on the internet using text mining. The natural language process includes word segmentation, word counting and counting the relationships between the words. A word cloud was developed, and clustering analyses were performed.

**Results:**

There were 19,565 posts about diabetes collected from forums, community websites, and Q&A websites in the summer (June, July, and August) of 2017. The three most popular aspects of diabetes were diet (33.2%), life adjustment (21.2%), and avoiding complications (15.6%). Most discussions about diabetes were negative. The negative/positive ratios of the top three aspects were avoiding complications (7.60), problem solving (4.08), and exercise (3.97). In terms of diet, the most popular topics were Chinese medicine and special diet therapy. In terms of life adjustment, financial issues, weight reduction, and a less painful glucometer were discussed the most. Furthermore, sexual dysfunction, neuropathy, nephropathy, and retinopathy were the most worrisome issues in avoiding complications. Using text mining, we found that people care most about sexual dysfunction. Health providers care about the benefits of exercise in diabetes care, but people are mostly concerned about sexual functioning.

**Conclusion:**

A conceptual gap between health providers and the network population existed in this real-world social media investigation. To spread healthy diabetic education concepts in the media, health providers might wish to provide more information related to the network population’s actual areas of concern, such as sexual function, Chinese medicine, and weight reduction.

**Supplementary Information:**

The online version contains supplementary material available at 10.1186/s12875-022-01846-0.

## Introduction

Diabetes is a common chronic disease; 451 million people worldwide had diabetes in 2017 [1], and 1.6 million deaths were directly attributed to diabetes in 2016 [2]. Diabetes can cause many complications in many parts of the body, including kidney failure, amputations, blindness, and nerve damage, and can increase the risk of mortality. Because of rapid urbanization and a sedentary lifestyle, the prevalence of diabetes has been increasing quickly over the past few decades [1, 2].

The main treatment for diabetes concentrates on controlling blood glucose, which is accomplished with diet, physical activity, and medication, as well as blood pressure and lipids to reduce acute or chronic complications. Blood glucose control in patients with diabetes involves mainly lifestyle adjustments, medical supervision, and self-management [3]. In fact, one study showed that the pressure on patients with diabetes comes mainly from four major issues: the ability to self-control blood glucose, the possibilities of future complications, the medical costs of controlling blood sugar, and a lack of support from family or friends [4]. Thus, many diabetes patients who fail to adhere to their treatment regimen will erode their health outcomes and increase the cost of medical care [5]. Thus, noncompliance is related to the “social distance” between doctors and patients, where the reasons for noncompliance include the failure of patients to understand their doctor’s advice, personal difficulties in life, and the economic capacity to follow medical advice [5].

In the past, to gain a deeper understanding of the problems related to blood glucose control among patients with diabetes, some qualitative studies used focus groups and in-depth interviews to obtain data for the research. However, these studies may not have accurately estimated the conditions of other patients with diabetes owing to the small number of samples and geographical limitations [6].

With the rise of social networks, the primary conduit for accessing medical information may be the internet rather than television, newspapers, and magazines. As online communities thrive, social networks amass a tremendous amount of discussion content. It is therefore possible to gain more direct insights into people’s thoughts about diabetes self-management in their daily lives. In online content, the respondent is more likely to have provided spontaneous answers (rather than passive answers) to relevant questions.

According to the “2017 Individual/Household Digital Opportunity Survey” conducted by the National Development Committee, the internet usage rate in Taiwan increased from 62.7% in 2005 to 82.3% in 2017 [7]. To better understand the various views of the general population on diabetes, unlike the previous methods used for qualitative research, this study mines big data from online communities to identify trending discussion topics relating to diabetes self-management among Taiwanese people.

It is expected that by analyzing spontaneous questions and discussions among the public, it will be possible to identify problems from a patient-centered perspective to provide references for diabetes health education or clinical care programs. This study has the following foci: 1. The distribution of the discussion content and the amount of the discussion about diabetes in/on social networks, discussion areas, and Q&A websites are observed, and the sentiments of people when discussing each aspect of diabetes are compared and categorized as positive, neutral, or negative. 2. After obtaining the three most discussed aspects, further observations are made of the words in the discussion that occur at higher frequencies, and relevant words in the discussion are presented through a word cloud.

## Method

### Research design and data sources

Data on diabetes self-management were collected and retrieved from social networks, and relevant information was analyzed and visualized with Chinese semantic analysis technology on the OpView social media observation platform [8] developed by Eland Information Co., Ltd. The data collection method employed was Directional Crawling (i.e., the data were collected from a specific source, and the data range was defined based on keywords), so the websites used as data sources were all-encompassing, i.e., approximately 20,000 channels from Taiwan famous discussion areas, such as main forums and well-known campus discussion areas (Dcard, etc.), social networks (Facebook fan pages, Plurk, Twitter, PTT, etc.), and Q&A websites (e.g., Yahoo! Answers). Relevant information was further filtered according to the main article or responses. We divided the range of data collection by season. The period for data collection was the summer (June, July, and August) of 2017.

To establish keywords on relevant topics and dimensions and further explore the trending discussion on each aspect using text mining to determine people’s views on diabetes, we used the aspects of diabetes patient self-care behavior modified from the AADE7 Self-Care Behaviors™, developed by the American Association of Diabetes Educators (AADE) for diabetes self-management education and training (DSME/T) and care, and the AADE7 Self-Care Behaviors® identified as essential for successful patient management [9]. These AADE7 aspects, i.e., diet, exercise, medications, blood sugar control, problem solving, avoiding complications, and life adjustment, were used to determine the aspects considered during data collection. Supplementary Table S1 shows the keywords related to the AADE7 aspects.

To avoid extracting content unrelated to the research topic “diabetes” and “high blood sugar,” we initially determined the quality of the data and excluded certain keywords (e.g., “pregnancy,” “gestation,” and other aspects or noise related to diabetes), as well as topics that were accompanied by too much advertising during the collection of the textual materials. We did not conduct further analyses until it was determined that the quality of the data met expectations.

We classified and individually stored the textual materials downloaded through the OpView social media observation platform according to each of the AADE7 aspects, compared the amount of discussion on each aspect through the R package ‘dplyr’ [10], and determined the distribution of relevant discussions of the different aspects on different social media platforms. For the textual materials downloaded on the OpView social media observation platform, the background management system of the OpView social media observation platform compared each article to the word banks and sentiment analysis by the Artificial Neural Network model and rated it as positive, negative, or neutral based on the corresponding documents or sentences. For the sentiment marking results, we compared people’s sentiments toward the different aspects under consideration and observed the general discussion related to each aspect. Supplementary Table S2 shows the sentiment scores of sentiment analysis.

By observing the general online discussion among the public concerning each of the AADE7 aspects, we used the “Sort by Popularity” for the article list, where the texts were segmented first and then transformed into the Document-Term Matrix (DTM), and identified the top three popularities of certain topics in the public discussions. After confirming whether the discussed topics were related to the aspects of interest, we excluded the content related to lucky-draw activities on Facebook fan pages and confirmed whether the discussed topics were related to specific aspects of interest. After a discussion with diabetes experts, the words were filtered and deleted to achieve dimension reduction. Finally, a word cloud was drawn and visualized on the WordArt website.

We used pointwise mutual information (PMI) [11] to measure the level of interdependence between words and to identify any co-occurrence of the words, where a higher value indicated a higher degree of co-occurrence between any two given words. We observed the distribution of the words in the texts concerning each aspect based on a hierarchical cluster analysis and determined the category for each word to further investigate their meanings. Cluster analysis was used to distinguish between specific aspects and to determine categories with high levels of between-group heterogeneity and low levels of within-group heterogeneity. We observed the level of importance of relevant words to certain aspects and provided relevant data for clinical physicians or nursing staff to refer to when they write prescriptions or provide health education to patients with diabetes.

## Results

### Data sources and the general discussion of each aspect

**Fig. 1:**
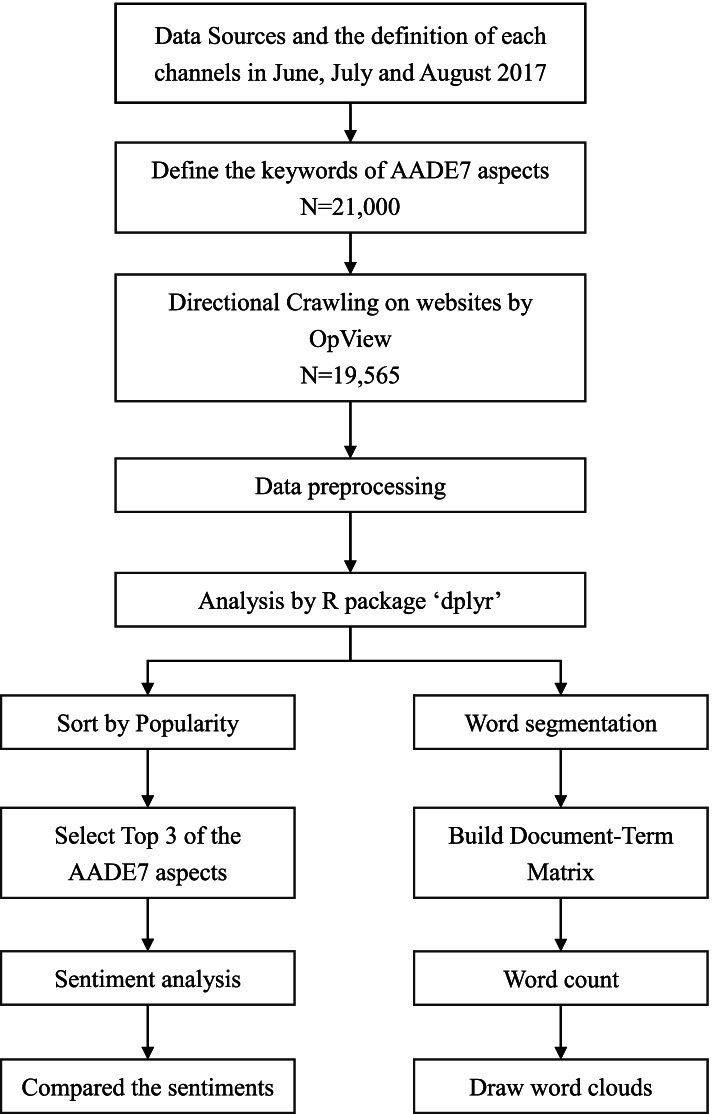
Research methodology flowchart

As shown in Fig. 1, we collected 21,000 data on the AADE7 aspects. After excluding certain keywords (e.g., “pregnancy,” “gestation,” and other aspects or noise related to diabetes), there were 19,565 data entries (including 4,181 posts and 15,384 comments), and the proportions were as follows: 33.2% for diet, 21.2% for life adjustment, 15.6% for avoiding complications, 11.2% for medications, 8.3% for problem solving, 6.2% for blood glucose control, and 3.2% for exercise. The distribution of the amount of discussion about topics concerning diabetes is shown in Table 1. Regarding the distribution of each aspect on the different social media outlets, diet was most discussed on social networks, exercise was most discussed on Q&A websites, medications/blood sugar control was most discussed on social networks and discussion forums, problem-solving was most discussed on discussion forums, avoiding complications was most discussed on Q&A websites, and life adjustment was most discussed on discussion forums. Overall, these aspects were most frequently discussed on discussion forums, where the proportion of relevant discussions amounted to 41.8%.

**Table 1:** The total amount of discussion for the AADE7 aspects of interest^a^

Aspect	All aspects n(%)	Diet n(%)	Life Adjustment n(%)	Avoiding Complications n(%)	Medications n(%)	Problem-Solving n(%)	Blood Glucose Control n(%)	Exercise n(%)
Social networks ^b^	7,512 (38.40%)	3549 (54.71%)	1000 (24.15%)	755 (24.19%)	991 (42.51%)	500 (30.64%)	500 (40.95%)	217 (34.28%)
Discussion areas ^c^	8,179 (41.80%)	2188 (33.73%)	2204 (53.24%)	890 (28.52%)	1000 (42.90%)	985 (60.36%)	638 (52.25%)	274 (43.29%)
Q&A websites ^d^	3,874 (19.80%)	750 (11.56%)	936 (22.61%)	1476 (47.29%)	340 (14.59%)	147 (9.01%)	83 (6.80%)	142 (22.43%)
Total Amount of Discussion	19,565 (100.00%)	6487 (33.20%)	4140 (21.20%)	3121 (15.60%)	2331 (11.20%)	1632 (8.30%)	1221 (6.20%)	633 (3.20%)
Rank		1	2	3	4	5	6	7

^a^modified by AADE7

^b^Social networks: Facebook fan pages, Plurk, Twitter, PTT, etc.

^c^Discussion areas: main forums, well-known campus discussion areas, Dcard, etc.

^d^Q&A websites: Yahoo! Answers

^e^Through the total number of posts and comments that mentioned the keywords we establish in each aspect, which reflects the degree of discussion, thus reflecting the “heated discussion”

**Fig. 2:**
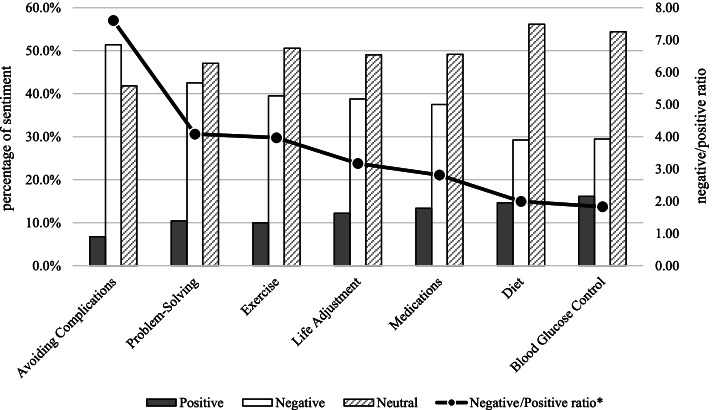
The sentiment (%) and negative/positive ratio for the AADE7 aspects. Y-axis: Left refers to the percentage of sentiment; Right refers to the negative/positive ratio

Regarding topics related to diabetes, we observed the sentiments toward each aspect and judged the sentiments toward the discussion content (positive, negative or neutral) to further understand the reactions of individuals when discussing the different aspects of interest in this work. Figure 2 shows that when members of the public discuss diabetes, they generally express more negative sentiments. When people see the articles shared by others, they have expectations and express a willingness to try recommendations. For example, the comment “It looks tasty! Even my diabetic father wants to try it!” shows a cheerful, grateful, and accepting sentiment, which makes the post positive overall. In contrast, complaints such as “I have diabetes, so I am not lucky enough to try it!” show sentiments such as grief, complaints, helplessness, and opposition, which makes the post negative overall. Among the AADE7 aspects, the most negativity was expressed in relation to avoiding complications had, with a negative/positive ratio of 7.60, followed by problem solving (4.08) and exercise (3.97).

### Topics accompanied by heated public discussions related to the top three aspects

The results show that the most discussed top three aspects over the internet were diet, life adjustment, and avoiding complications. Using “Sort by Popularity,” we identified the main topics discussed by the public in relation to the top three aspects to understand the public’s views and concerns about these specific aspects. The contents of the top three aspects are discussed below. The contents of the other four aspects are shown in Supplementary Table S3.

#### Diet

In heated discussions by the public concerning diet, the topics centered on seeking folk remedies, special diet therapies, and Chinese herbal medicine prescriptions.

Individuals attempted to find folk remedies based on ancient recipes and relevant herbs and ingredients on the internet to determine the best way to control their blood sugar.

“What Chinese herbal medicine can lower blood sugar?”

The general population was happy to share how to use folk remedies or ingredients that could control blood sugar, as long as the suggestions were found to be useful and effective.

“My father has been drinking bitter melon water. It is quite effective. We usually use 3-4 bitter gourds to cook a pot of bitter melon water. The seeds are also immersed in the water. Of course, we must continue to take the prescribed medicine, but the bitter melon water should help control the blood sugar better.”

Another topic of concern among the public was the use of special diet therapies to help stabilize blood sugar. Popular topics include the Low-Carb diet and the Low-GI diet.

“My father is also a diabetic patient. I myself am a dietitian, but I do not recommend the Ketogenic Diet or a Low-Carb Diet without careful consideration because I am more concerned about the more serious harm of shock from low blood sugar. However, I would still recommend the Low-GI Diet.”

Some people also mentioned that their blood sugar level was not stable even though they had a habit of engaging in exercise and took their medicine regularly. Even if they have been taking Western medicine over a long period of time, it is highly likely that they will seek help from a Chinese herbalist.

“...My father has diabetes and cardiovascular diseases, and his condition has worsened even though he has been taking Western medicine for a long time. My father found a Chinese herbalist doctor and took the Chinese herbal medicine for nearly 2 years. He was cured by the Chinese herbalist doctor with the Chinese herbal medicine. During the past 2 years, we’ve tracked all data related to his body conditions, and all data have been normal...”

#### Life adjustment

When faced with diabetes, many people cannot adjust their mood because of the dramatic changes occurring in their bodies. They may complain a lot and become resentful and frustrated from the moment they know about their illness and while receiving treatment, taking medicine, controlling their blood sugar, etc. Family members can also be emotionally affected.

“In my hometown, my grandpa has had diabetes for 30 years. He was never been cured. Everyone knows that what a problem diabetes is. Patients with diabetes can be perfectly normal in the normal course of life. But when they suddenly have a big meal, their blood sugar level can skyrocket and make everyone rush off their feet!”

Economic issues are a major burden for many people with diabetes and their families. For example, a female netizen said that her husband is the pillar of the economy in her family, but owing to the disease, he has failed to bring in income, and the economic pressure has made the woman want to divorce her husband.

“My husband has had diabetes for 4 months, and he hasn’t had any income for 4 months…

I have to work and pay the mortgages and utility bills on my own. I just want to divorce…”

Some people also mentioned that since the elders in their families have suffered from diabetes with complications, they have incurred considerable medical expenses, so they were concerned about their future economic position.

“My father passed away six months ago. He is gone, and my money is gone. If you want to ask me what I can do now, I really have no idea.”

#### Avoiding complications

In heated discussion concerning avoiding complications, topics centered on sexual dysfunction, diabetic neuropathy, diabetic nephropathy, and diabetic retinopathy.

Among the many potential issues that can be caused by diabetes, sexual dysfunction is an issue of much concern among the public. As it can be difficult to talk about this problem, people turn to the internet for answers.

“How to cure sexual dysfunction caused by diabetes?”

“How to treat erectile dysfunction without medication?”

“How to treat sexual functioning caused by diabetes?”

They may use the internet to determine whether they can improve this problem using drugs. People often recommend medicine that they think is useful.

“Sexual dysfunction can be caused by diabetes. To tackle this disease, strict control over the blood sugar is of utmost importance.

*I have also been troubled by diabetes for a long time, and my sexual desires have dwindled. I am engaging in exercise and taking bitter gourd peptide capsules to control my blood sugar. “*

The general population is worried because they do not know how to identify complications following a stroke, and they do not know which department to consult for therapies.

*“Hello to everyone on the Tainan board. My mother has diabetes. During the past two days, she said that she felt numb over the top knuckle of her index finger. She thought it would be all right to just rub the top knuckle. However, last night, she started to feel nothing. She was afraid that diabetes had caused nerve necrosis. Therefore, I want to ask which doctor or clinic I can seek help from.”*

Meanwhile, in the face of seasonal changes, there are concerns about whether the large difference between the indoor and outdoor temperatures will cause headaches and peripheral neuropathy as people with diabetes enter and exit an air-conditioned room.

“Can patients with diabetes stay in an air-conditioned room? In summer, people generally need more air conditioning.

I’ve heard that the difference between indoor and outdoor temperatures will not only make people sick easily but will also cause peripheral neuropathy.”

Since long-term medications are required for people with diabetes to control their blood sugar, people are concerned about whether the use of blood sugar-lowering medicine will cause lesions to the kidney.

“Will the long-term use of blood sugar/blood lipid-lowering medicine by patients with diabetes cause any lesions in the kidney/liver?”

Some people may not understand the complications of diabetes very well, so they only discover that they have serious lesions in their eyes when they go to the hospital.

*“My father has had diabetes for more than 10 years, and he was never told to go through any ocular fundus examination. He could see things clearly, and he never felt any discomfort in his eyes. However, only through the examination was it discovered that his left eye had been stricken by severe retinopathy.”*

### Locate content that is potentially of concern among the public through text mining

In this study, textual materials concerning the top three aspects (diet, life adjustment, and avoiding complications) were segmented, and the frequency of each word was calculated. Then, clinical physicians selected key words representing each aspect, and these words were visualized via word clouds (Supplementary Figure S1). An association analysis was then conducted on the selected words to pinpoint the words with a high degree of co-occurrence. Finally, cluster analysis was conducted on each aspect to calculate the results of the classifications.

In the word cloud for the diet aspect, when individuals are seeking folk remedies and recipes, they may mention words such as “Chinese medicine” and “nutrition.” For special diet therapies, they may often mention “ketone,” “fat,” “weight reduction,” etc. “Chinese herbalist doctor” is also mentioned to indicate concern about Chinese herbalist doctors. In the word cloud for the aspect of life adjustment, words such as “sex,” “pressure,” “illness,” “control,” “exercise,” and “insulin” are frequently mentioned. In the word cloud for avoiding complications, words such as “sex,” “dysfunction,” “central nerve,” “erection,” and “hypertension” are frequently mentioned. Regarding complications, sexual dysfunction is a frequently discussed topic.

Additionally, in the word cloud for the avoiding complications aspect, words such as “blood vessels,” “kidney,” “nerves,” “infections” and “heart disease” also reflect that the public is concerned about diabetes-related complications, such as diabetic nephropathy, dialysis, diabetic neuropathy, poor wound healing, and diabetic cardiovascular diseases.

Through a word frequency count, we selected words that frequently appear in the texts, i.e., words with correlation values equal to or above 4.0, based on calculations using R. In the diet aspect, the word correlations showed that in regard to Chinese herbalist doctors, words such as prescriptions, therapeutic effects, and how to enhance sexual function (dysfunction) were also moderately correlated and were a major concern among individuals. In the life adjustment aspect, words related to sex may indicate that people are troubled by sexual dysfunction. They may also show the trouble people are experiencing in this area. Pressure is highly correlated with words such as “obesity” and “fat.” This correlation might well indicate concerns regarding obesity. Males are highly correlated with words such as “sex,” “sexual function” and “dysfunction,” which may reflect concerns related to sexual dysfunction caused by diabetes. Meanwhile, combinations of highly associated words, such as “kidney + deficiency” or “stroke + dementia”, may also indicate problems encountered by individuals in this regard.

By observing topics that involve heated discussion and analytical methods such as word frequency counts, association analyses, and cluster analyses, we can generate insights into the three aspects of interest, namely, diet, life adjustment, and avoiding complications, in terms of different analytical methods shown in Table 2.

**Table 2:** Content of the top three aspects emerging from the qualitative analysis and text mining

**Aspect**	**Qualitative Analysis**		**Text Mining**		**Summary**^a^
	**Content of Topics of Heated Discussion**	**Word Cloud**	**Word Association Analysis**	**Cluster Analysis**	
**Diet** Total Amount of Discussion 6487	1. Seeking help from folk remedies/Chinese herbal medicine to control blood sugar levels 2. Using special diet therapies to improve dietary choices and stabilize blood sugar 3. When the effects of western medicine are limited, people will seek help from Chinese herbalist doctors	The Word Cloud showed words and frequencies, such as: Chinese herbalist doctor (522), nutrition (250), fat (217) and weight reduction (151), indicating that the public may frequently mention Chinese herbalist doctors, diet -related nutrition issues, and obesity problems	From the associations between words, was discovered that the public want to try the Ketogenic Diet. When a Chinese herbalist doctor is mentioned, the prescriptions, therapeutic effects, and how to enhance the sexual function (dysfunction) may also be the major concern among the public	From the two major clusters in terms of diet, it was found that the public may care about how their diet can be combined with medications to help control blood sugar levels, and they want to understand the nutritional value of food to help stabilize their blood sugar levels	People are more concerned about types of diets, nutritional values, and Chinese herbal medicine, and they mainly hope that they can obtain relevant information to improve their condition
**Life Adjustment** Total Amount of Discussion 4140	1. If a diabetic patient is emotionally unstable, his/her family members will also be affected 2. Medical expenses to treat diabetes are a huge burden on a nuclear family 3. When choosing a blood sugar meter, people often focus on how the blood sugar meter can reduce pain and minimize sounds	The Word Cloud showed words and frequencies, such as: sex (2915), pressure (2263), illness (2000), control (1476) and lesion (584), indicating that the public frequently mentions problems about sex, blood sugar control, and many other illnesses that may be associated with life pressure	Words related to sex may show the trouble faced by this population, such as sexual dysfunction. Pressure is highly correlated to words like obesity or fat. This might well indicate the concerns of the public about obesity	From the three major Life Adjustment aspects, it was found that the public may suffer pressure from problems caused by complications of diabetes and obesity, and they may need to inquire about blood sugar control methods suggested by physicians	The major sources of life pressure among the public may arise from sexual dysfunction caused by diabetes and weight reduction related to obesity
**Avoiding Complications** Total Amount of Discussion 3121	1. Concern about complications of diabetes and other issues, such as sexual dysfunction, caused by diabetes 2. Concern about whether there are stroke symptoms or not and the headaches caused by the differences between indoor and outdoor temperatures in summer 3. Concerned about diabetic nephropathy due to long-term use of blood pressure-lowering medicine 4. Misunderstandings about complications have caused serious diabetic retinopathy among patients with diabetes	The Word Cloud showed words and frequencies, such as: sex (18,220), dysfunction (950), central nerve (603), erection (538), kidney (156), nerve (136), and heart disease (125), indicating that the public frequently mentions words related to sex and problems about the nervous system, kidney, and cardiovascular disease	Males are highly correlated to words like sex, sex function and dysfunction, which may well reflect the concern of the public about sexual dysfunction caused by diabetes. Meanwhile, combinations of highly associated words, such as kidney + deficiency or stroke + dementia, may also indicate problems encountered by the public	From the three major clusters of complications, it was found that the public may suffer mental pressure from issues specific to men. Meanwhile, sexual dysfunction is clearly of great concern, and people are very concerned about lesions in their bodies	This population is mostly concerned about sexual dysfunction, diabetic nephropathy, cardiovascular diseases, and stroke

^a^Summary: A summarized form the four approaches

Regarding the diet aspect, by observing the content of speech, it was found that people mainly discuss folk remedies or special diet therapies that may help control blood glucose levels. When the effect of Western medicine is limited, people will seek help from Chinese herbalists.

Analysis based on text mining showed that the words frequently appearing on public discussion forums are related mostly to nutritional values and the type of diet. Relevant words also appear in public discussions about Chinese herbalist doctors. The analytical results are quite consistent with the results of direct observation of the textual materials.

The results of the life adjustment aspect are somewhat different. By observing the content of the topics in discussion forums, it was found that discussion focused on the diabetic patient’s influence on the family’s mood or even the economic burden caused by the diabetic patient to the family.

Meanwhile, the choices and use of a blood sugar meter also led to heated discussions. However, through the analysis based on text mining, it was found that most words were related to sexual function (dysfunction) and concerns about obesity. These analytical results were somewhat different from the results from the direct observation of the textual materials.

In the avoiding complications aspect, the discussion concerned mainly sexual dysfunction caused by diabetes, diabetic nephropathy caused by long-term use of medicine, diabetic neuropathy caused by seasonal changes, or diabetic retinopathy caused by ignorance and misunderstanding of diabetes.

However, analysis based on text mining showed that the public is most concerned about sexual function (dysfunction). Meanwhile, words such as diabetic nephropathy and cardiovascular diseases also appeared in their discussions. The analysis of the textual materials and results of text mining indicated that the public is most concerned about sexual dysfunction in regard to avoiding complications.

## Discussion

After observing the topics of concern to individuals, it was found that clinical foci are different from what the public truly cares about. For example, although diabetes health education and advice from physicians generally emphasize exercise [12], the amount of discussion about exercise over the internet was the lowest among the AADE7 aspects of self-care behavior, indicating that exercise is of little concern to the public. Meanwhile, in terms of health education for people with diabetes or prescriptions, clinical physicians rarely mention issues concerning diabetes-related sexual function (dysfunction). However, in public discussions, it is the most frequently mentioned issue. Additionally, regarding obesity, clinical physicians promote the importance of weight reduction [13], which is also a topic of much concern to the public.

The results of this study indicate that people express more positive sentiment when discussing the most discussed aspects related to diet. This finding is similar to a study in which 260 Post-It notes were collected and divided into 123 concepts and 24 clusters. The most frequently mentioned words were eating, negative sentiment, and the complications of diabetes [14].

Therefore, the results of this study may be somewhat different from the results of past studies. Many previous studies have mentioned that people with diabetes often do not know what to do about their diet because they fail to grasp the seriousness of their blood glucose levels and many other complications [12, 13, 15]. However, the results of this study indicated that diet is discussed most on public forums. In the diet aspect, the public is concerned about folk remedies, special diet therapies, or Chinese herbal medicines that can help control blood glucose levels. The results of text mining show that nutritional values, therapeutic effects, how to improve blood glucose through diet, and how to reduce fat intake are mentioned the most. The following changes in sentiment were observed after discussing the diet aspect: when people obtain more information on diets for diabetes over the internet, such as folk remedies, cooking methods, and dietary options, they feel they have more solutions, and they thus feel more confident about their diet. Thus, they tend to be more positive. By investigating factors that may cause heated discussions about specific topics among the public, it was found that the discussed topics must contain specific and clear topics/content to arouse interest. Such concepts may be subsequently applied in health education and informational propaganda since they provide relevant information about specific themes discussed on social media platforms that will help expel doubts and help form correct ideas about diabetes. This phenomenon also echoes social influence theory [16, 17]. Nonetheless, it is worthwhile to further study correlations between the sentiments of people discussing certain issues and the information obtained to determine whether there is a causal relationship in the data over time.

To investigate factors that cause negativity and lower the degree of concern among the public when discussing exercise, we observed content of discussion and found that people are less motivated to engage in exercise mainly because they feel that it has limited effects that are not immediately perceivable or because they have a busy life and have difficulty maintaining exercise habits. Establishing a habit of exercise or increasing the amount of exercise is often regarded as one of the major challenges faced by patients with diabetes [3]. We refer to a study on factors hampering self-management among patients with diabetes from Portugal in 2015, which found that difficulties related to exercise mentioned by patients include lack of motivation and willpower and failure to foster the habit. The study also mentioned that fatigue and muscle and joint pain, among other complications, such as heart disease and foot disease, can make exercise even more difficult [18]. In 2017, another study was conducted in India to investigate factors hampering the cultivation of an exercise habit based on a qualitative analysis [19]. The results of the study pointed out factors hampering the cultivation of an exercise habit among participants, such as lack of time, insufficient health awareness, little support from the equipment or environment, gender inequality, limitations on physical strength, and physician ignorance. These factors made it difficult for patients to engage in routine exercise even though they truly wanted to, which is quite similar to the results obtained in the present study.

Similarly, the results of performing text mining and word associations showed that males and words such as sexual dysfunction were highly correlated. The results of the cluster analysis showed that pressure on men and sexual function (dysfunction) were significantly related. Both the observation/discussions and the text mining results indicated that the public is concerned about sexual function (dysfunction) when they talk about diabetes-related complications. The reason for this finding may be that people are shy when visiting a doctor, and it is difficult to ask an attending physician such questions, so they seek answers on the internet. A 2012 U.S. study on the willingness of patients to discuss self-care behavior with physicians [20] found that although most patients think confessions to physicians are very important for the treatment process, nearly 1/3 of patients are not willing to discuss issues related to the self-care process with physicians. Therefore, one study suggests that treatments should be based on trust between health providers and patients, where a friendly, open-minded conversation can bridge this gap [21].

The text mining for potential messages concerning the life adjustment aspect showed that the public often mentions words such as males and sexual function (dysfunction) and concerns about obesity and being fat, which are sources of stress in their life. As speculated in this study, the potential reason for these concerns may be that weight reduction is not easy for people already suffering from obesity. For people with diabetes, weight reduction may not be easy because they may be older, suffer from other complications, engage in less exercise than their younger counterparts, or take other blood sugar/lipid-lowering medicine that causes them to gain weight easily.

In this study, the content of public discussions showed that when an article is marked with positive sentiment, it shows that the writer of the article is in a cheerful mood and wants to share a useful method with other netizens who have the same problems. However, when an article is marked with negative sentiment, it shows problems concerning blood glucose control, which leads to complaints or sentiments of sadness and confusion. For example, a diabetic patient may feel helpless because he or she wants to try a special diet but cannot do so owing to objections from the family, or a diabetic patient may be dubious about the potential hazards from methods shared by other netizens and thus may be skeptical that it may cause more risks. Even communication from family members can be affected by the emotional instability of elderly individuals suffering from diabetes and may lead to complaints. As we carefully observed the sentiment markers for each article, we found that the sentiments may not be opposing, so the classification criteria for neutral and positive sentiments may not be obvious. Thus, it is worthwhile to consider how to improve the sentiment determination method.

Since the data in this study are meant for research use by OpView-sponsored academic institutions, the time frame for data collection was quite limited. In this study, the data were divided temporarily by season to focus on three months (June, July, and August) in the summer of 2017. In the future, if the time range can be extended, observations of the changes in the amount of online discussion about diabetes and the general situation over the long term would be insightful. Since the textual materials for analysis were from open data sources, the sociodemographic variables could not be controlled exactly and thus cannot be inferred in detail. Meanwhile, through the OpView social media observation platform, the data collected via the keywords did not clearly distinguish between type 1 and type 2 diabetes, and it was impossible to distinguish whether the content was posted by people with diabetes or their family members.

Regarding the sentiments toward the discussion content, each article is marked with a positive, negative, or neutral sentiment by the OpView social media observation platform, which compares words in the word banks and conducts calculations based on historical data and the machine learning method. However, after scrutinizing the markup of each text in detail, we found that the results may not be perfect. In addition, in text mining, only the associated words can be listed based on the association rules, so the reasons for the high relevance cannot be confirmed. The data in this study were all from social networking platforms. The ideas of these community users may not represent the opinions of the general public. At present, most community users are still young, which is the main limitation of the present study. Nonetheless, big data available from online communities can save the time and money; although they still only partially represent the opinions of the public, they can eliminate regional limitations more than has been possible in the past. Therefore, the main bias is that we could not identify who posted the content, but we were able to ensure the person was associated with or suffered from diabetes. The data sources of this study are primarily interactive platforms, which gather messages from the public who post information, answer questions, and discuss topics. Rather than merely being able to comprehend the opinions of patients in the past, it is now feasible to comprehend a broader range of perspectives.

By observing the topics of concern among the public, it was found that clinical foci may be different from the concerns of the public. From the social media content, it was possible to determine how much the public is concerned about the importance of self-care behavior for diabetes. The results showed that the topics of greatest concern were diet, life adjustment, and avoiding complications, which are somewhat different from the general content of concern in clinical medicine. Through emerging technology based on big data obtained from online communities, this study points to information asymmetry between health care providers and the public, identifies diabetes-related myths and key factors of primary concern, and provides insight into how these messages and topics affect individual sentiments and the established concepts in the minds of the public so that health care providers can find corresponding forms of communication and design better diabetes self-management education programs to tackle relevant issues.

In summary, if research is to break through these limitations, in segmentation of words in textual materials, different word banks could be cited for analysis and comparison, or a custom dictionary could be added to improve the results of word segmentation. Subsequent interpretations can also be completed and could be made more incisive. Thus, with regard to establishing topics, keywords can be more accurate. For example, insulin, the glycemic index (GI), and other concepts about diabetes could be added to the analytical framework.

Regarding the content of concern among the public, such as sexual dysfunction caused by diabetes and obesity, it is suggested that relevant themes and health information be published on social media platforms to expel doubts in the minds of individuals. Therefore, future research should consider the frequency at which information about sexual function (dysfunction) caused by diabetes should be published on various social media platforms to strengthen the concept of “preventive medicine” and promote self-health management among a younger population. Even though exercise did not appear to be a public concern, it is an important concern of health providers, so online platforms can be used to promote the idea of routine exercise, for example, by using the slogan “More exercise leads to better sexual function.” Diabetes education should be based on the content of concern. There is still much room for big data from online communities to be applied in clinical medicine. For instance, the theme of diabetes in this study can be extended to address more details, such as the condition of people taking blood-glucose-lowering medicine, the high penetration rate of the blood glucose meter, and the user experience. In addition, an exploration of different diseases, such as dementia and osteoporosis, may be required to gain a deeper understanding of how the public perceives different diseases, thus providing a more appropriate approach to health education.

In the future, elderly people will also tend to increase their network influence. Although this study uses keyword settings to collect textual materials, when investigating the content of discussion about diabetes self-management, it was not possible to clearly identify whether the relevant content was the patient’s own opinion or the view of the patient’s family members. Nonetheless, this study provides relevant contributions and leads to a clearer understanding of what Taiwanese people think about diabetes.

It is well known that health providers care about blood sugar levels, compliance with medication, and adverse drug effects in the clinical context. However, patients care about diet and alternative medicine as well. In addition, they care about complications, especially sexual dysfunction. Therefore, from this study, diabetes education about diabetes self-management policies should be changed to focus on the concerns of people with diabetes and their social world. In conclusion, reducing the gap between health providers and the general population is the only way to promote positive outcomes related to diabetes self-management.

## Supplementary Information

**Additional file 1:** **Supplementary Table S1.** The keywords related to the AADE7 aspects. **Supplementary Table S2.** The sentiment scores of Sentiment analysis. **Supplementary Table S3.** The content of the other four aspects. **Supplementary Figure S1.** Word cloud analysis of the top three aspects of interest.

## Data Availability

The datasets used and/or analyzed during the current study available from the corresponding author on reasonable request.

## Abbreviations

AADE: American Association of Diabetes Educators
DSME/T: Diabetes self-management education and training
DTM: Document-Term Matrix
