# Supplementary material for: Using social media data in diabetes care: bridging the conceptual gap between health providers and the network population
Source: BMC Prim Care. 2022 Sep 17;23:241. doi: 10.1186/s12875-022-01846-0 (PMC9482184; doi:10.1186/s12875-022-01846-0)
Supplement: Supplementary file 1 — Additional file 1: Supplementary Table S1. The keywords related to the AADE7 aspects. Supplementary Table S2. The sentiment scores of Sentiment analysis. Supplementary Table S3. The content of the other four aspects. Supplementary Figure S1. Word cloud analysis of the top three aspects of interest. [file 12875_2022_1846_MOESM1_ESM.docx]

**Supplementary Table S1. The keywords related to the AADE7 aspects**

| **AADE7 aspects** | **Keywords** |
| --- | --- |
| Diet | Diet, eating, drinking, food, meal, portion, nutrition, supplement, calories, rice, vegetables, meat, bowl, oil, snacks, protein, milk, fruit, staple food, treat, wedding banquet, flavoring, fat, etc. |
| Exercise | Exercise, aerobic, anaerobic, walking, jogging, running, mountain climbing, cycling, swimming, yoga, Tai Chi, Wai-Tan-Kung, morning exercises, jogging, sports, exercise habits, etc. |
| Medications | Medicine, insulin, needle, medicine, secret prescription, folk prescription, treatment, therapy, oral, long-acting, short-acting, doctor, etc. |
| Blood glucose control | Blood glucose, hba1c, a1c, blood glucose measurement, target, blood glucose measurement, record, etc. |
| Problem-solving | Problem, unsatisfactory, uncomfortable, bad, myth, error, treatment, method, solution, why, will it go wrong, precautions, how to do, etc. |
| Avoiding complications | Complications, causes, lesions, sequelae, consequences, heart, blindness, amputation, obstruction, perception, blood vessels, nerves, sexual function, infection, wounds, feet, sensations, pain, etc. |
| Life adjustment | Life, adjustment, care, small tips, pressure, achievement, frustration, adjustment, discussion, bothering, trouble, worry, method, reference, information, etc. |

**Supplementary Table S2.** **The sentiment scores of** **Sentiment analysis**

The sentiment is scored from the database of OpView platform, using the Artificial Neural Network model to identify positive, negative, or neutral in those documents or meaning of sentences which been crawled from web. The words had been segmented, and identified individual words in those documents with respect to different sentiment scores. We continuously expanded the maturity of the model and the richness of the vocabulary through model training and vocabulary matching.

Positive and negative sentiment scores are calculated according to the following formulas separately, with a score between 0 and 1.

$$Positive sentiment score=\frac{Numbers of positive in those documents by Manual Tagging}{Numbers of positive in those documents by Auto \mathrm{tagging}}$$

$$\mathrm{Negative} sentiment score=\frac{Numbers of \mathrm{negative} in those documents by Manual Tagging}{Numbers of \mathrm{negative} in those documents by Auto \mathrm{tagging}}$$

When the positive score of an article exceeds 0.3, the article is judged as significant positive sentiment; when the negative score of an article exceeds 0.3, the article is judged as significant negative sentiment; When both the positive and negative scores of an article less than or equal to 0.3, the article is judged as neutral.

|  | Positive | Neutral | Negative |
| --- | --- | --- | --- |
| Sentiment scale | Positive score >0.3 | Both positive and negative scores <=0.3 | Negative score >0.3 |
| Words | best, easy to use, effective | not bad, not really | sad, disappointed, too bad, dislike |

**Supplementary Table S3. The content of the other four aspects**

| **Aspect** | **Content** |
| --- | --- |
| **Medications**  (Total Amount of Discussion 2331) | - I have a wound but I don't know how to apply the medicine   *“I probably started to have wounds in early May. Because I had diabetes, the wounds rot very quickly. The doctor said that blood sugar will be used as nutrient. I thought it was just a small wound and applied for the medicine casually.”*   - High cost of insulin creates financial burden   *“I just can collect recyclable materials and trade them for some cash to barely make ends meet. I really can't pay for my child's insulin fees and medical expenses at the same time.”*   - Diabetic patient refuses to take medication   *“My mother was diagnosed with diabetes. The doctor prescribed medicine for her, but she said she should not take medicine or see a doctor again. What will happen if she does not take medicine? How can I persuade her?” she is very stubborn.*   - Side effects of diabetes medicine   *“After taking diabetes medicine, I feel pain and weakness in the right half of my body, diarrhea and abdominal bloating.”* |
| **Problem-Solving**  (Total Amount of Discussion 1632) | - Understand how to check for the symptoms and the causes of diabetes   *“My family medical history had diabetes, but my parents and I currently don't have it. If I want to see a doctor, should I an appointment with Nephrology?”*  *“I would like to ask is Diabetes an incurable disease, how to prevent and what are the causes?”*   - How to care for people with diabetes at home   *“My mother is almost 60 years old and looks healthy. Although she has diabetes, she is under stable control (drug & diet control). What else should I pay attention to?”* |
| **Blood Glucose Control**  (Total Amount of Discussion 1221) | - Questions about Blood Glucose Control   *“My grandfather had diabetes for more than 30 years, and it was incurable. It is no problem in normal life. As long as you suddenly eat a big meal and your blood sugar soars, everyone will be upset.”*  *“If the blood sugar is too high and will get diabetes, can stop being diabetic by lowering the blood sugar or is it an incurable chronic disease?”*   - Questions about Blood Glucose testing   *“A blood sugar of 126 after 8 hours fasting is a diagnosis of diabetes. How many people in Taiwan have a blood sugar over 126?”* |
| **Exercise**  (Total Amount of Discussion 633) | - Exercise with limited effects or lazy   *“The efficiency of exercise is too low. Running for an hour a day will only lose one kilogram a month, but drinking a sports drink after exercising will add one kilogram at once.”*  *“My grandfather has diabetes and he is not exercising, so his health has never been very good. Once he went out to buy things, he suddenly fainted (it seems to be related to lower blood sugar) and he could not walk very well after being hospitalized. He is very lazy to do the rehabilitation.”* |

1. Diet

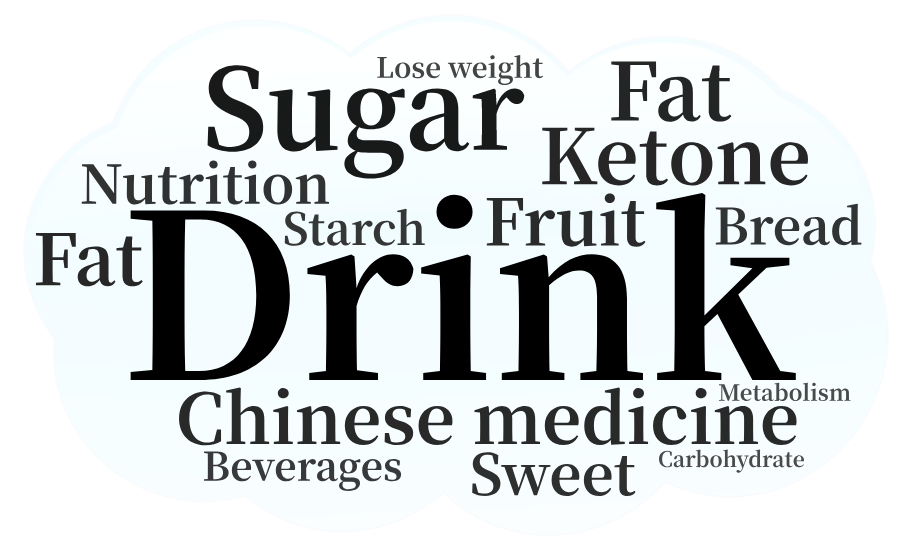

2. Life Adjustment

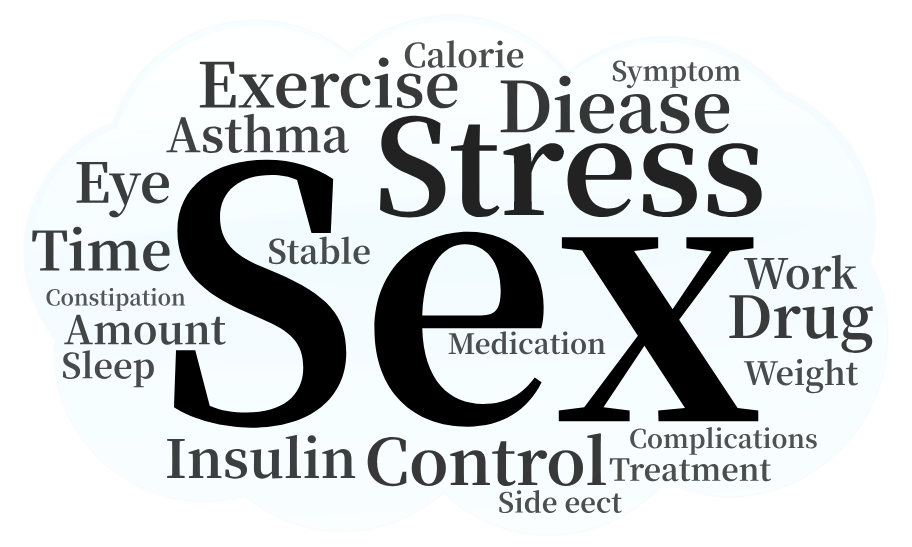

3. Avoiding Complications

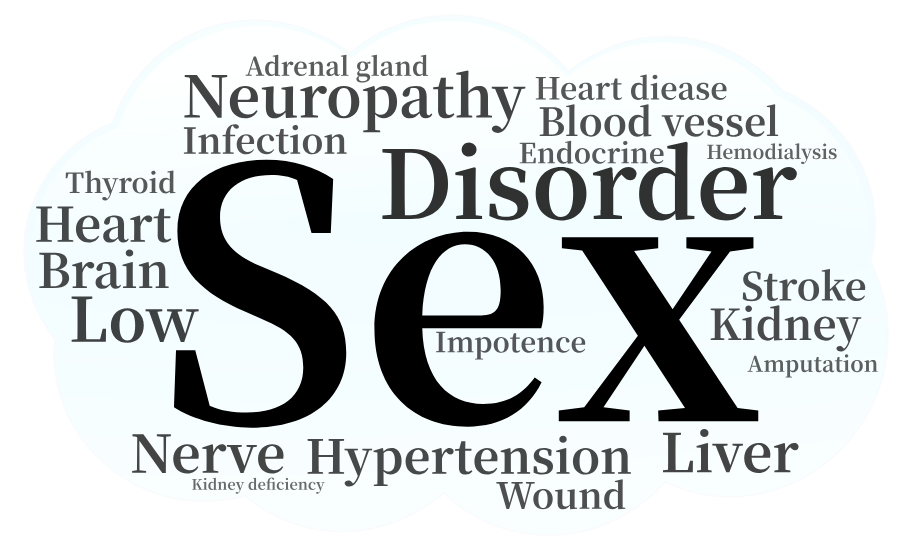


**Supplementary Figure S1. Word cloud analysis of the top three aspects of interest.**
